# Face Presentation, Mento-Posterior Position

**Published:** 1884-04

**Authors:** 


### Face Presentation, Mento-Posterior Position.

At a recent meeting of the Medical Club, Dr. M. D. Mann reported a case of labor with the head in the mento-posterior position, in which the patient, being fully anæsthetized, he succeeded in bringing down the vertex, thus converting the exceedingly untoward position into one entirely favorable. This rather unusual result was accomplished by means of one hand in the vagina, pressing up the chin and face, while the other hand, placed externally over the pubic region, was employed in pushing down the occiput. The unfavorable position, however, continued to repeat itself at each subsidence of the anæsthetic condition, and was as often corrected by the doctor, until at last the delivery was accomplished by applying the forceps while holding the head in its favorable position. We may also notice that the Tarnier axis-traction forceps, recently described in the JOURNAL, as also in the recent work of Lusk, was employed in the earlier stage of the delivery. During the discussion, the possibility of natural delivery, with the head remaining in the mento-posterior position, was denied, but as will be seen from the following annotations, such cases have been reported and the mechanism variously explained.

Cazeaux says ("Theoretical and Practical Midwifery," pages 341-2):

1. The rotation just described, whose object is to bring the chin constantly towards the symphysis pubis, and which has been spoken of as absolutely essential to the spontaneous termination of the labor, may not be executed. But such very rare exceptions do not in the least discredit the general principle before laid down, for they may all be referred to those instances where the dimensions of the head are small relatively to those of the pelvis; or else to those cases in which the position of the face has been spontaneously converted into one of the vertex. True, Madame Lachapelle has known the face to escape from the vulva in a transverse direction, or nearly so, in two or three instances; but she carefully adds that they were very rare exceptions.

And again (pages 343-4-5):

2. As regards those varieties in which the chin looks backwards, we have already stated that it is necessary this part should come round in front, though some cases of mento-posterior positions, that terminated spontaneously, are found in the books, where the chin did not get under the pubic arch; writers differ in their explanations of this anomaly. M. Velpeau takes as an illustration the mento-sacral variety, or the second position of Baudelocque, in which the chin is turned toward the anterior face of the sacrum (though we may observe, in passing, that this position is scarcely admissible); and he remarks that, as the chin does not rotate in front, the following phenomena may then take place: the forehead engages behind the body or the symphysis of the pubis, while at the same time the chin gets below the sacro-vertebral angle. The whole head descends into the excavation beyond the anterior plane, and the face drags after it the front surface of the neck, and even the upper part of the chest behind. The occipito-mental diameter, which still represents the axis of the strait very nearly, now begins to perform a see-saw movement from above downwards, and from behind forwards. The chin, penetrating further and further towards the bottom of the excavation, though at the same time retained by the thorax, which cannot advance, forces the sagittal suture to slip down behind the pubis, and the forehead to gain the upper part of the inferior strait. The frontal protuberances soon find a point of resistance on the perinæum, and the posterior fontanelle descends in turn, and ultimately appears at the summit of the arch, when the head finally escapes from the vulva as it would in an occipito-anterior position: whence it follows, adds M. Velpeau, that the *occipito-frontal is the greatest diameter* which can present at the planes of the strait. But we cannot admit the truth of this last proposition; for if, as he says, the chin is in relation with the anterior surface of the sacrum, and it *descends* more and more, while the occiput slips behind the pubis, it is evident that the *occipito-mental* diameter must, at a given moment, traverse the antero-posterior one of the excavation. Now, as this is clearly impossible, we have to reject M. Velpeau's explanation altogether. Beside, the cases observed by Smellie and Delamotte, which he cites in support of his theory, prove nothing at all, for, in both of those instances, the fœtuses were small and dead, and the woman had, on former occasions, been delivered of voluminous children.

M. Guillemot has explained the spontaneous termination of the labor in these cases somewhat differently; for when the chin does not rotate in front, the labor, according to his idea, may terminate in two ways, namely: 1st. The forehead continues to descend and to engage under the branch of the pubis until the anterior fontanelle appears at the vulva, which progression permits the chin to advance forward and reach the border of the perinæum; then the process of flexion commences, etc. But we cannot conceive how, in the forced extension of the head on the thorax, it is possible for the chin to arrive at the anterior perineal commissure by traversing the whole posterior plane of the excavation, because, from all evidence, the breast must engage extensively along with the head, which is wholly impossible, unless it be a case of abortion.

2d. The labor by the face may be converted into one by the vertex; and this always takes place, he continues, in the following manner: the face being forcibly pressed on, and unable to escape through the perineal strait, has a natural tendency to pass towards those points that offer the least resistance. Here, this condition is found above and behind, whence the chin leaves the perineum and approaches the fœtal chest by ascending along the hollow of the sacrum towards the sacro-vertebral angle, and the forehead following this movement corresponds to the sacrum in turn; the vertex is depressed and slips behind the pubis, and, just at the moment when the chin applies itself to the child's breast, the occiput engages under the pubic arch. He further supposes the face to be sufficiently engaged for the chin to come in contact with the perineum; but, as we have already stated, this is impossible, on account of the extent of the conjoint diameters of the head and breast, both of which would be deeply engaged in the excavation.

But, even admitting the chin should descend so low, where is the power to make it subsequently rise up in the hollow of the sacrum, the cavity of which is occupied, whatever M. Guillemot may say to the contrary, by the deeply engaged breast? For the uterine contraction, which is always transmitted by the spine, acts at first on the chin as a consequence of the reverted position of the head (as M. Velpeau clearly recognized), and it is only because its power is adequate to make the latter descend any further, that its action is transferred to the other extremity of the fronto-mental diameter, that is, to the forehead, which it then depresses, according to the theory of Guillemot. Again, even supposing that the chin may remount, it is scarcely possible to believe that it gets above the sacro-vertebral angle; it must, therefore, constantly remain in contact with the anterior suface of the sacrum; and, consequently, at a given moment, the occipito-mental diameter must traverse the antero-posterior one of the excavation.

In my estimation, therefore, we are not to understand this as the true mode by which the mento-posterior positions of the face are converted into occipito-pubic ones; indeed, among all the cases I have been able to consult, I have only found there in which the chin was in direct relation with the anterior face of the sacrum, viz., those of Smellie, Delamotte and Meza (reported by Guillemot).

Now, in the one furnished by Smellie, it is positively stated that the child was small, that the woman had a large pelvis, and that she was usually delivered very promptly. Delamotte says nothing about the head and the dimensions of the pelvis, in his case; and lastly, Meza was obliged to apply the forceps, in the one reported by him; of course, that was no longer a spontaneous termination, for it would be an easy matter to demonstrate that the application of the forceps may act in an altogether different manner, and even more advantageously, than the uterine contraction in this instance; besides, the reader will not forget that, in the first two cases, the children came away dead.

All the other observations may be referred either to the right or the left mento-sacro-iliac positions; and, in these latter, it appears to me that a spontanous termination of the labor might occur without a simultaneous engagement of the chest and head; for instance, let us suppose that the child is in the right mento-sacro-iliac position; then, after the complete extension of the head, the face will descend into the excavation as far as the length of the neck permits, and consequently the chin will reach the level of the great sciatic notch, the more so as the form of this portion of the ilium, which is shaped like a cone, will favor the movement of downward progression. Having arrived at this notch, the chin will there encounter soft parts, which it can very readily depress, and this depression will be quite sufficient to augment the length of the oblique diameter of the excavation from a quarter to a half an inch, thereby permitting the occipito-mental diameter to clear it, and the head to undergo the process of flexion, that will gradually bring the occiput under the pubic symphysis.

Robert Barnes ("Obstetric Operations") remarks as follows: "The birth of a full-grown living or recently dead child, with the forehead maintaining its direction forward, is almost impossible. The extension of the neck is extreme, the head being doubled back upon the nucha. The chin represents the apex of a wedge, the base of which is formed by the forehead, the entire length of the head, and the thickness of the neck and chest. This must be equal to at least seven inches. The vagina and occiput become flattened in, it is true, but much is not to be expected from moulding." Regarding the method of delivery and the means of aid, he says: "In the mento-sacral position you deliver by promoting flexion; or, to take our illustration from the mechanism of face labor, you obtain flexion by causing the chin to turn over the coccyx or sacro-sciatic ligament as a center instead of over the symphysis. The latter is the natural mode, but it may be that the first alone is possible. This is a case in which incision bilateral of the perinæum here acting as an obstructing posterior valve, may be performed in order to facilitate the release of the chin." Lusk, in his work on the "Science and Art of Midwifery," says: "In a fœtus of small size the face may, when it meets with slight resistance from the perinæum, be born in any of the pelvic diameters. Instances of spontaneous delivery without anterior rotation of the chin are, however, extremely rare. He then proceeds to give his opinion as being that "at full term the face presenting spontaneous delivery in mento-posterior position is not practicable." He adds that "it is claimed, however, that when the head is small and compressible it may stretch either the sacro-sciatic ligaments when oblique, or the perinæum, after passing the extremity of the sacrum to an extent sufficient to permit the descent of the occiput beneath the pubic arch, and the conversion of the face into a vertex presentation." He also adds that "Smellie, Hicks and Braun, of Vienna, have, however, each reported a case of forceps delivery by drawing the chin down over the sacrum and perinæum, when the occiput and calvarium glided underneath the pubes." He also mentions two similar cases reported by Dr. I. E. Taylor.

Playfair notices the subject in the following words: "In order fully to understand the subject, it is necessary to study those rare cases in which the chin points backwards, and forward rotation does not occur. These may be taken to correspond with the occiput posterior position, in which the face is born looking to the pubes, but, unlike them, it is only very exceptional that delivery can be naturally completed. The reason of this is obvious, for the occiput gets jammed behind the pubes, and there is no space for the fronto-mental diameter to pass the anterior-posterior diameter of the outlet. Cases are indeed recorded in which delivery has been effected with the chin looking posteriorly, but there is every reason to believe that this can only happen when the head is either unusually small or the pelvis unusually large. In such cases the forehead is pressed down until a portion appears at the ostium vagina; when it becomes firmly fixed behind the pubes, and the chin, after many efforts, slips over the perinæum. When this is effected, flexion occurs, and the occiput is expelled without difficulty. The forehead is probably always on a lower level than the chin. Dr. Hicks has published a paper in which he attempts to show that this termination of face presentation is not so rare as is generally supposed, and he gives a single instance in which he effected delivery with the forceps, but he practically admits that special conditions are necessary, such as the antero-posterior diameter of the outlet particularly ample, and a diminished size of the head. When delivery is effected it is probable, as Cazeaux has pointed out, that the face lies in the oblique diameter of the outlet, and that the chin depresses the soft structures at the side of the sacro-sciatic notch, which yield to the extent of a quarter inch or more, and thereby permits the passage of the occipito-mental diameter of the head. It must, however, be borne well in mind that spontaneous delivery in mento-posterior position is the rare exception, and that supposing rotation does not occur—and it often does so at the last moments—artificial aid, in one form or another, will be almost certainly required."

This article, having already attained considerable length, the similar notices of Bedford, Glisam, Leishman and others, which we have taken time to look up, are omitted. Sufficient have been quoted to prove that while the cases are extremely rare, they serve, like so many others in medicine, to show that there is hardly a rule without its exceptions.

Since writing the above we learn that Dr. Hauenstein, of this city, delivered with forceps in a case of face presentation, mento-posterior position. This occurred some twenty years ago.

